# Cellulose based hydrogel as soil conditioner and seed germination medium

**DOI:** 10.1038/s41598-025-05920-2

**Published:** 2025-07-02

**Authors:** Shu-Jun Jong, Suk-Fun Chin, Mohd Effendi Wasli

**Affiliations:** Faculty of Resource Science and Technology, University of Malaysia Sarawak, 94300 Kota Samarahan, Sarawak Malaysia

**Keywords:** Cellulose-based hydrogel, Plasticiser, Soil moisture, Seed germination media, Chemistry, Materials science, Biomaterials, Soft materials

## Abstract

To address the research gap concerning the effects of different plasticisers on the properties of cellulose hydrogel, this study investigated the impact of various gelling agents, including sodium carboxymethylcellulose (NaCMC), alginate (AA), glycerol, and polyethylene glycol (PEG), on cellulose-based hydrogels. Among these plasticisers, hydrogels containing 1.75% (wt/wt) NaCMC, 1.0% (wt/wt) glycerol, and 0.5% (wt/wt) PEG exhibited excellent water uptake and retention capacities, absorbing over 2600% of water and retaining at least 10% of that water over a 96-h period. These cellulose hydrogels also improved the soil moisture content, pH, and electrical conductivity across four different soil types (sand, topsoil, gley soil, and clayey soil) over a 15-day period. Additionally, the hydrogels were assessed as seed germination media for Choy Sum vegetable seeds. The study demonstrated that glycerol-plasticized cellulose hydrogel significantly enhanced seed germination, even without an external water supply, due to its superior water retention properties. Biodegradability tests revealed that all hydrogels lost over 80% of their weight after 60 days in loamy soil, confirming their biodegradability.

## Introduction

Soil properties are essential for plant survival, with soil moisture content being a key factor. Insufficient water content in the soil can hinder the survival of many plants. Currently, the moisture content of lots of land has decreased due to the effect of climate change. This has led to significant water loss for many crops, making them unable to survive due to unfavourable environmental conditions. As a result, crop production in developing countries was severely impacted, leading to a disruption of food security^1^. Recently, hydrogels have garnered significant interest from researchers due to their potential to enhance agricultural development and address the challenges faced by crops in affected countries^2^. A hydrogel is a three-dimensional structure of hydrophilic polymers composed in a cross-linked network. The hydrophilic functional groups in the hydrogel primarily interact with water molecules, which enhances its ability to absorb large amounts of water^3^. Cellulose-based hydrogel is widely used in many studies due to its abundant hydroxyl groups, which enhance its water retention and functional properties^3–5^. Wastepaper is an ideal starting material for producing cellulose-based hydrogel because it is a renewable and sustainable biomass.

Plasticisers, also known as gelling agents, play a crucial role in the formation of cellulose hydrogel by enhancing its flexibility and malleability. This, in turn, helps prevent cracking and increases the hydrogel’s elasticity^6,7^. The influence of glycerol and PEG as the plasticiser in hydrogel has been studied in recent years. PEG plasticised in the hydrogel improved the thermal stability and the polymerisation effect of the water in the hydrogel because the flexibility of the polymer chains increased in the hydrogel^8,9^. Besides, the reduction of the density of the crosslinked network in the hydrogel has caused the high swelling capacity and the water retention ability of the hydrogel found in the hydrogel incorporated with PEG^10^. The hydrogel incorporated with glycerol has also demonstrated considerable strength and flexibility^11^. Apart from their benefits in hydrogel formation, PEG and glycerol as plasticisers also present some drawbacks. Increasing the amount of PEG and glycerol will directly affect the cross-linking density, which will make the network in the hydrogel more compact and detrimental to their swelling ability^5,12^. Besides PEG and glycerol, sodium carboxymethylcellulose (NaCMC) is also a commonly used plasticiser in hydrogel formulations. The hydrogel plasticised with the optimal amount of NaCMC exhibited improved swelling due to the elastic network’s refraction force. However, a higher concentration of NaCMC in the hydrogel can negatively impact the flexibility of the crosslinking networks, similar to the drawbacks associated with PEG and glycerol^13–16^. On the other hand, there are few studies on the effect of alginate (AA) on the properties of hydrogels. Although alginate can serve as a gelling agent, most studies only used alginate as a starting material to prepare alginate-based hydrogels for pharmaceutical and biomedical applications, mainly due to their high costs^17^.

There is not much research that compares the effects of these four plasticisers on the hydrogel properties. Therefore, in this study, the cellulose hydrogels underwent a gelation process with four plasticisers (sodium carboxymethylcellulose (NaCMC), alginic acid (AA), glycerol, and polyethylene glycol (PEG)) with Epichlorohydrin (ECH) as the crosslinker. Cellulose hydrogel was plasticised with four different plasticisers, namely alginic acid (AA), sodium carboxymethylcellulose (NaCMC), glycerol, and polyethylene glycol (PEG) with different amounts (0.50–2.50 wt%) to determine the optimal concentration of each that would enhance surface morphology, water absorption capacity, and water retention capacity. Additionally, cellulose-based hydrogels with optimal water absorption and retention were selected to study their effects as soil amendments on different soil types, including sand, topsoil, gley soil, and clayey soil. This study also evaluated the performance of these cellulose hydrogels as seed germination media for Choy Sum vegetable seeds, as well as their biodegradability.

## Experimental

### Materials

The wastepaper was collected from the Faculty of Resource and Science Technology’s office at the University of Malaysia Sarawak (UNIMAS). Sodium hydroxide (NaOH), and 95% ethanol were purchased from HmbG® Chemicals (Germany). The plasticisers used in the formation of the hydrogel are alginic acid (AA), sodium carboxymethyl cellulose (NaCMC), glycerol, and polyethylene glycol (PEG) were purchased from Acros Organics (New Jersey, United States), Sigma-Aldrich (Darmstadt, Germany), and R&M Chemicals (Essex, United Kingdom). Epichlorohydrin was purchased from Sigma-Aldrich (Darmstadt, Germany). All the chemicals were used without further purification. The four soil types used in this study were sand, topsoil, gley soil, and clayed soil. The vegetable seeds Choy Sum (brand: Framoc) were used in the seed germination assay on various types of cellulose hydrogels.

### Extraction of cellulose from wastepaper

The wastepaper was soaked in deionised water until it became pulp. The pulp was soaked in the 12% (wt/wt) of NaOH solution for 24 h to remove the ink, lignin and hemicellulose in the wastepaper^14,18^. After 24 h, the cellulose was washed with water and 95% ethanol. The pre-treated cellulose was dried in an oven at 60 °C until all the water was removed. Once dried, the cellulose fibres were ground and then filtered through a sieve.

### Synthesis of cellulose-based hydrogel

The NaOH/urea solvent with the ratio of 7:12 per 100 mL was precooled in the freezer until the temperature of the solvent reached − 12 °C^3^. The 3 g of cellulose fibre was dissolved in the pre-cooled NU solvent and then frozen for 12 h. After 12 h, the mixture was thawed and stirred until a homogeneous solution was formed. The gelling agents (alginic acid (AA), Sodium carboxymethyl cellulose (NaCMC), glycerol, and polyethylene glycol (PEG)) with variety concentrations (0.50–2.50 wt%) were then added to the solution respectively with continuous stirring. Epichlorohydrin (ECH) was added dropwise to the mixture and sonicated for 30 min. The resulting hydrogel was then stored in the freezer.

### Characterization of cellulose-based hydrogels

Each sample’s Fourier transform infrared (FTIR) spectra were obtained using a FTIR Spectrometer, Thermo Scientific Nicolet iS 10 (United States). The surface morphology of each cellulose-based hydrogel with AA, NaCMC, glycerol, and PEG was observed using the Field Emission Scanning Electron Microscope (FESEM) (LEO, 1525). The specific surface area, average pore size, and pore volume of hydrogels were analysed using the BET Surface Area Analyzer (Quantachrome Autosorb iQ-AG) based on nitrogen gas sorption at 77 K and Quantachrome AS1Win software (Quantachrome Instruments Version 2.01)^19^.

### Water uptake capacity of cellulose-based hydrogels

To determine the swelling ratio of each cellulose hydrogel with different concentrations of gelling agents of AA, NaCMC, glycerol, and PEG (ranging from 0.50 to 2.50 wt%), the dried samples were first weighed, and their weight was recorded before being immersed in distilled water. Every hour, the weight of each swollen sample was measured and recorded. Equation (1) was then used to calculate the water uptake capacity of the hydrogel:

1 $${\text{Water}}\;{\text{uptake}}\;{\text{capacity}} = \frac{{{\text{W}}_{{\text{s}}} - {\text{W}}_{{\text{d}}} }}{{{\text{W}}_{{\text{d}}} }} \times {1}00\% ,$$

where ${\text{W}}_{{\text{s}}}$ is the weight of swollen hydrogel while ${\text{W}}_{{\text{d}}}$ is the weight of the dried hydrogel.

### Water retention rate of cellulose-based hydrogels

To assess and compare the water retention capacity of the cellulose hydrogel with varying concentrations of gelling agents (AA, NaCMC, glycerol, and PEG) from 0.50 to 2.50 wt%, each of the swollen hydrogels was left at room temperature by keeping the swollen samples in the open air for the specified duration. Equation (2) was used for calculating the water retention rate:

2 $${\text{Water retention rate}} = \frac{{\left( {{\text{W}}_{{\text{t}}} - {\text{W}}_{{\text{d}}} } \right)}}{{\left( {{\text{W}}_{{\text{s}}} - {\text{W}}_{{\text{d}}} } \right)}}{ } \times 100{\text{\% ,}}$$

where ${\text{W}}_{{\text{t}}}$ is the weight of the wet hydrogel at the designed time; ${\text{W}}_{{\text{d }}}$ is the weight of the dry hydrogel; ${\text{W}}_{{\text{s}}}$ is the weight of the swollen hydrogels at room temperature.

### Assessment as a soil condition

#### Soil moisture of various soil types

Four types of soil (sandy soil, topsoil, gley soil, and clayey soil) from the Real Living Lab at Universiti Malaysia Sarawak (UNIMAS) were used in this study. All the soils were dried under the sun to dehydrate them before being used. After the soils were dried, the soils were sieved to remove debris, such as branches, stones, and root systems. Next, the soil moisture of each dried soil was measured using the digital soil moisture meter (Extech Instruments-MO750) to ensure the soil was fully dehydrated. Next, 50% of each swollen cellulose hydrogel, with different plasticisers and excellent performances in water uptake capacity and water retention rate, was mixed with 50% of each soil type for one minute before being placed into 6 cm × 6 cm × 6 cm planting bags. This was done to ensure all hydrogels were evenly mixed with the soil. The soil moisture of each sample was measured at the designated time using a soil moisture meter (Extech Instruments-MO750). The soil moisture of each sample was measured three times at three different marked points in each planting bag, and the average value was used for analysis.

#### pH and electrical conductivity of the soils

At the designated time, soil samples from each planting bag were collected into centrifuge tubes, and water was added to each tube in a 1:5 ratio. After adding the water, the samples were shaken for one minute to ensure thorough mixing of the soil and water. The samples’ pH and electrical conductivity were measured using a pH/conductivity meter (Cole-Parmer PC100) over a period of 15 days. The pH and electrical conductivity of each sample were measured three times at three different locations and the average of the readings was recorded.

### Seed germination assay

This study also determined the seed germination rate of Choy Sum (*Brassica chinensis var. parachinensis*) vegetable seeds in hydrogels with different types of plasticisers. The Choy Sum (*Brassica chinensis var. parachinensis*) vegetable seeds were used for planting in the hydrogel. The seeds were soaked in water for 4 h before use. A wetted cotton pad was used as the base for the control set of this experiment. Twenty seeds were placed in the control set and on each type of hydrogel in the Petri dishes, which were then kept at room temperature for a month without recording the humidity. Two sets of seeds were planted directly on the cotton, each with different parameters (one requires daily watering, and one does not). The seeds germinating on the hydrogels were not watered throughout the experiment. Seed germination under each condition was observed and recorded daily until the end of the observation period. The germination rates were calculated based on the International Seed Testing Association (ISTA, 1985) using Eq. (3):

3 $${\text{Germination}}\;{\text{Rate}}\left( \% \right) = \frac{a}{b} \times 100\%$$

where a is the total number of germinated seeds and b is the total number of seeds used.

Besides the germination rate of the Choy Sum vegetable seeds, the germination value (GV) was calculated using Eq. (4):

4 $${\text{Germination}}\;{\text{Value}} = \frac{{\left( {{\text{sum}}\;{\text{of}}\;{\text{the}}\;{\text{DGS}}} \right)\left( {\text{N}} \right)}}{{\left( {\frac{{{\text{final}}\;{\text{cumulative}}\;{\text{germination}}\;{\text{rate}}}}{10}} \right)}}$$

where DGS and N are the daily germination speed and the number of daily counts.

### Biodegradability of cellulose-based hydrogels

To assess the biodegradability of the cellulose-based hydrogel with different gelling agents, the following method was employed in this study^20^. The hydrogels were weighed and recorded before being buried in the loamy soil (a balance of sand, silt and clay soil) as known as the garden soil. Then, the weighted hydrogels were buried 5 to 10 cm deep in the loamy soil. The compost materials adhering to the surface of the hydrogel were removed using a brush before weighing the mass of the hydrogels after a certain period. The experiment was concluded when the weight of the samples stabilised and no longer changed. Equation (5) was used to calculate the weight loss of the hydrogel through the biodegradability test.

5 $${\text{Weight}}\;{\text{loss}}\left( \% \right) = \frac{{W_{o} - W_{i} }}{{W_{o} }} \times 100\%$$

where $W_{o}$ is the initial weight of the hydrogel and $W_{i}$ is the weight of hydrogel after interval time.

#### Statistical analysis

In order to improve the accuracy of the experiment data, all the experiments above, such as water uptake capacity, water retention rate, soil moisture, soil pH, soil electrical conductivity and biodegradability of each hydrogel, were carried out in triplicate, and the seed germination was carried out in quintuplicate. The experimental data were analysed using ANOVA, with statistical significance set at p ≤ 0.05. Consequently, the results of each experiment were represented as mean ± standard deviation.

## Results and discussion

### FTIR analysis of cellulose-based hydrogels

**Fig. 1:**
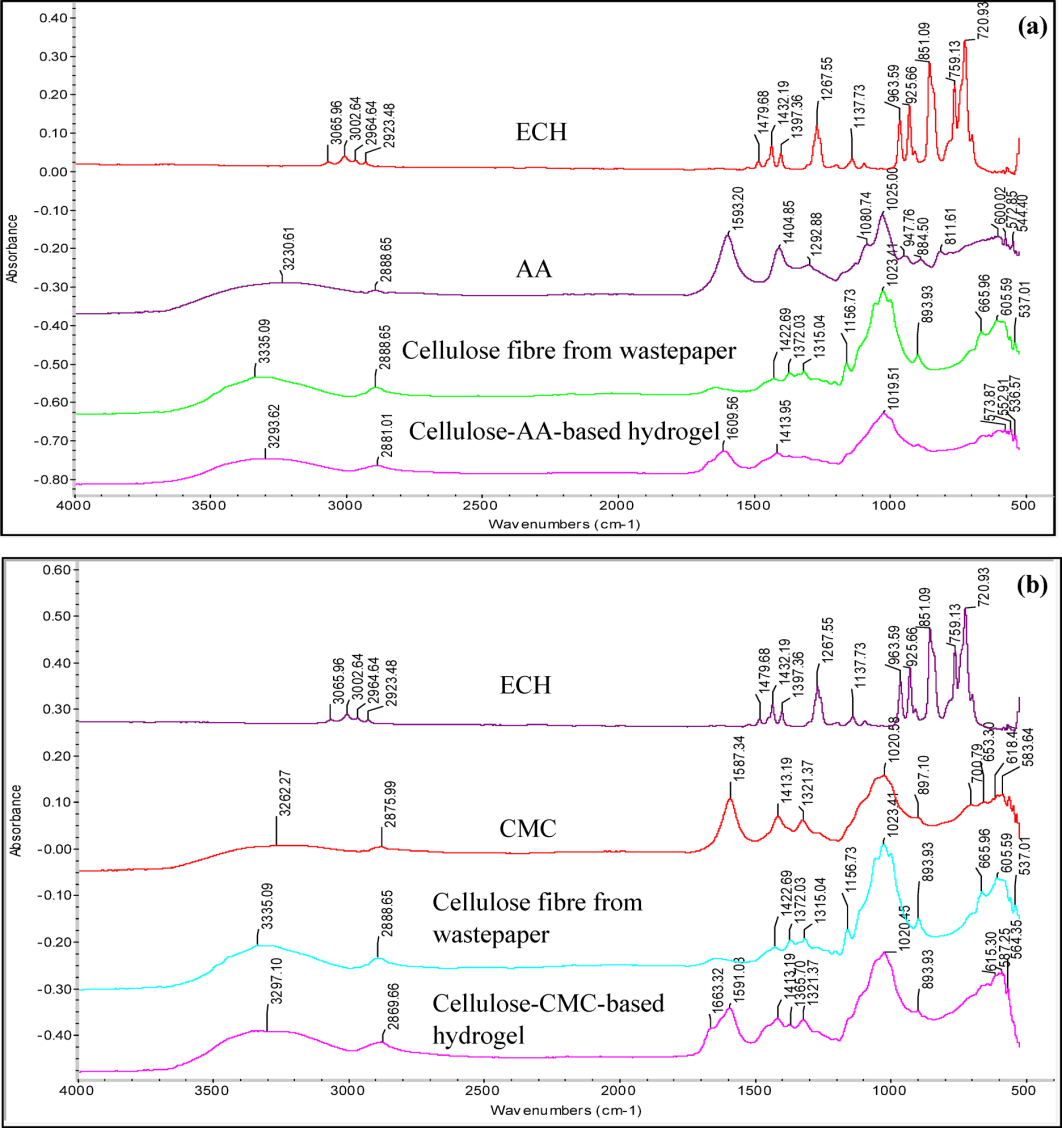

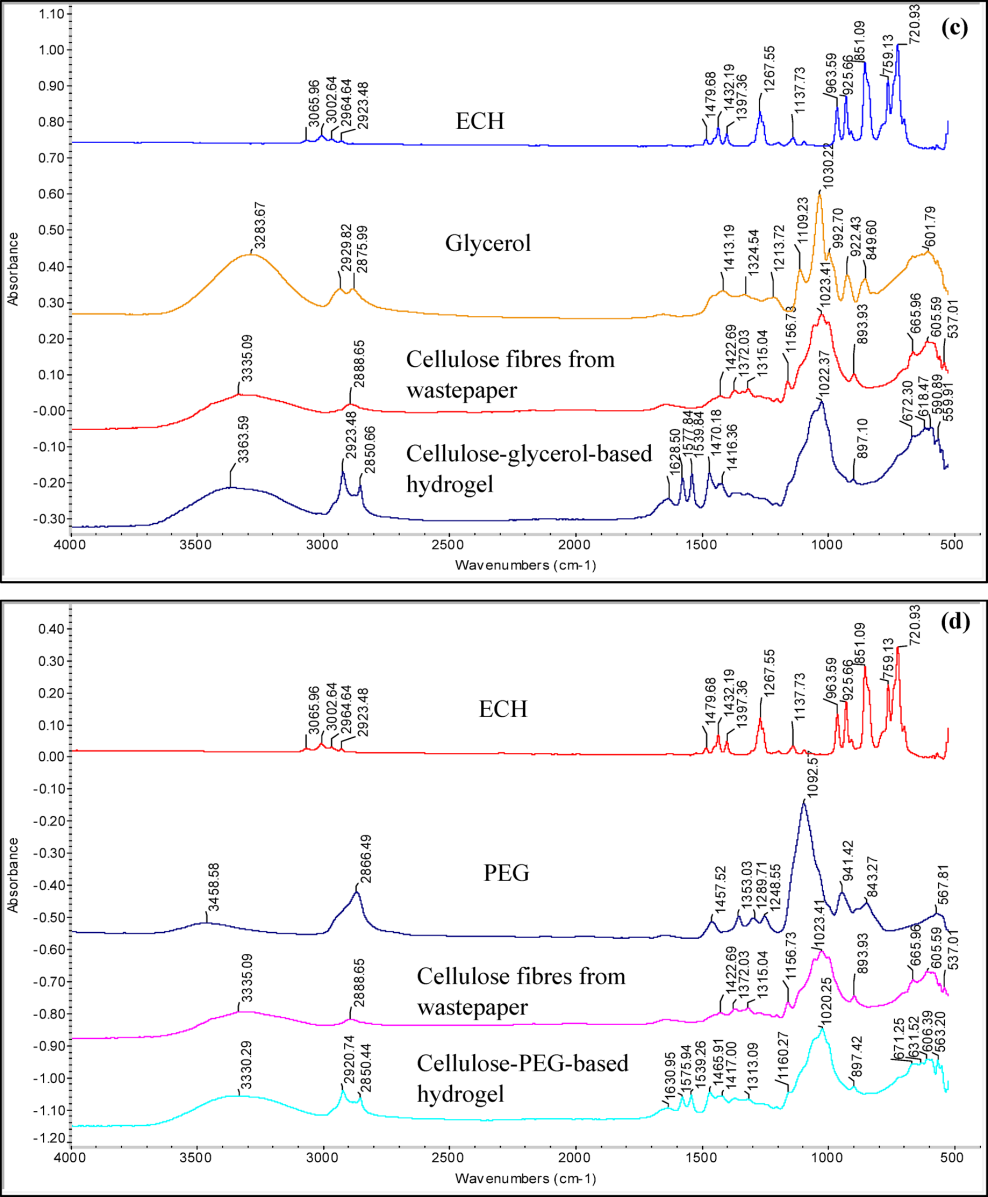
The FTIR spectra of (**a**) cellulose-AA-based hydrogel, (**b**) cellulose-CMC-based hydrogel, (**c**) cellulose-glycerol-based hydrogel, and (**d**) cellulose-PEG-based hydrogel.

The spectra of all the hydrogels in Fig. 1a–d, plasticised with four different plasticisers, display –OH stretching bands at around 3200 cm^−1^ to 3365 cm^−1^. This peak is important for all the hydrogel because this indicates the -OH stretching had successfully formed with the cellulose chains either self-association or entanglement of cellulose chains through the hydroxyl groups with the help of ECH^3^. Additionally, the hydroxyl functional groups present in all the hydrogels contribute to strong intermolecular hydrogen bonding within the hydrogels’ structure at around 3293 cm^−1^ and above. The hydrogel plasticised with glycerol in Fig. 1c had the stronger –OH stretching bonding evidenced by the 3363.59 cm^−1^ which has a higher wavelength than the other hydrogels. This indicates the plasticising effect of glycerol on the cellulose chains, which is attributed to the hydrophilic nature of both glycerol and cellulose^21^. Based on the FTIR spectra of all the hydrogels, OH bonding plays a crucial role in the structure of the cellulose chains, significantly contributing to the hydrogels’ water-absorbing capacity.

Moreover, the carboxyl groups that bonded with the cellulose chains in the structure of the cellulose-NaCMC-based hydrogel in Fig. 1b are at the wavelength of 1591.03 cm^−1^ in its spectra^22^. The spectra of cellulose-AA-based hydrogels at around 1609 cm^−1^ and this peak represented the symmetric vibrations of carboxyl groups^23^. The peak at 1465 to 1470 cm^−1^ in the hydrogels with glycerol and PEG indicated the presence of -COOH. Additionally, the spectra of the cellulose-glycerol-based hydrogel at 2923 cm⁻^1^ and 2850 cm⁻^1^ correspond to the C-H stretching vibrations. The peaks observed at 2929 cm⁻^1^ and 2875 cm⁻^1^ in glycerol shifted to lower wavelengths in the hydrogel.^24^. Furthermore, the peak observed at 1365 cm⁻^1^ in the cellulose hydrogel plasticised with NaCMC confirms that the crosslinking process was successfully carried out, indicating the formation of C–O–C bonds between the polymer chains^13^. The peak of all hydrogel samples between 1413 and 1417 cm^−1^ also belongs to the stretching vibration of COO − asymmetric^13^.

Besides, the FTIR spectra of the cellulose-glycerol-based hydrogel exhibited peaks at 2923 cm^−1^ and 2850 cm^−1^ are the C–H stretching, indicating the presence of the glycerol in the hydrogel^24^. In addition, the peak of 2850 cm^−1^ in the spectra of the hydrogel plasticised with PEG in Fig. 1d corresponds to the symmetric stretching vibration of CH₂^25^.

### Water uptake capacity of cellulose-based hydrogels

**Fig. 2:**
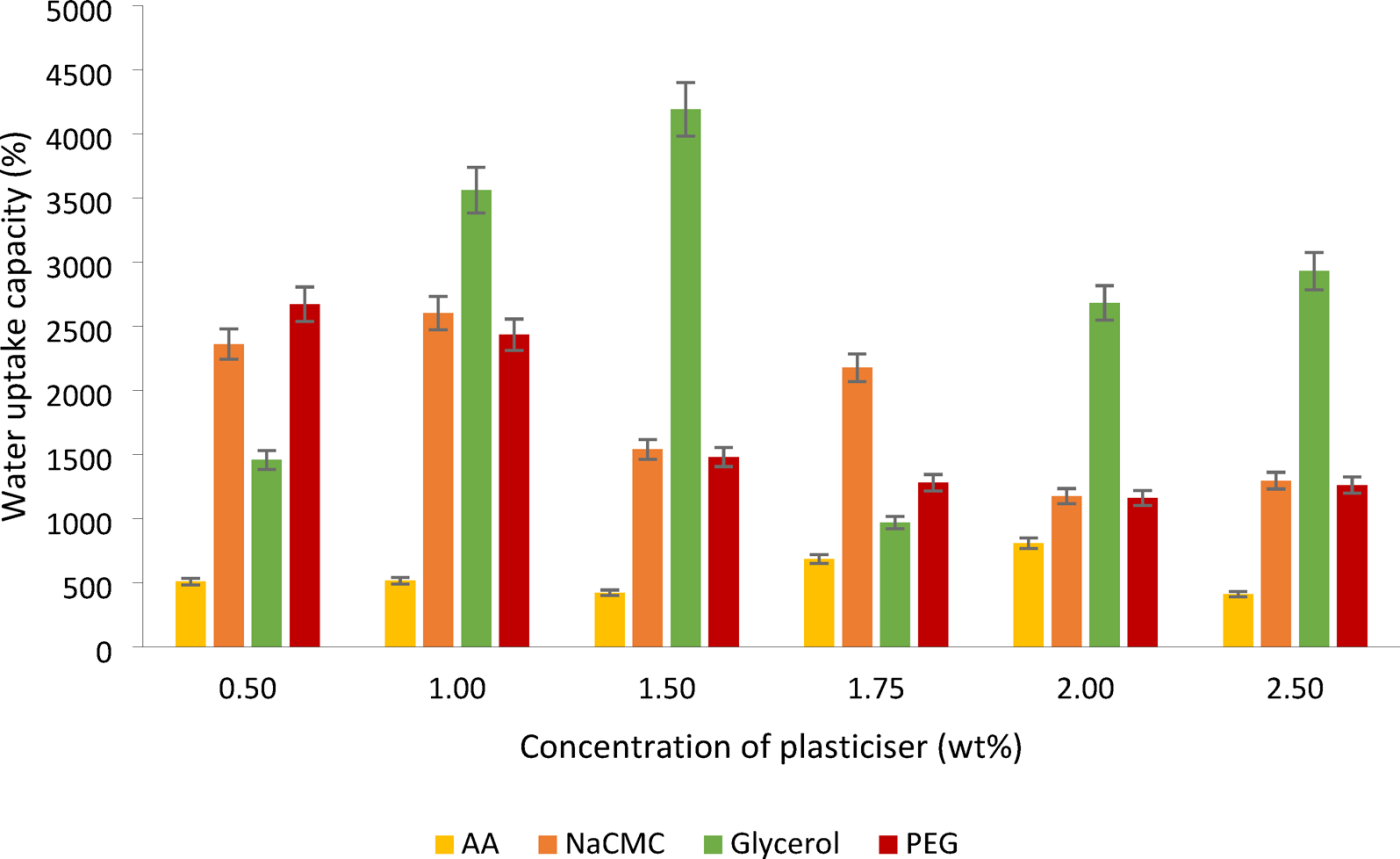
The swelling ratio of the cellulose-based hydrogel with varying amounts of plasticisers after 5 h.

As shown in Fig. 2, after 5 h, the cellulose-glycerol-based hydrogel exhibited the highest water uptake capacity compared to the other cellulose-based hydrogels plasticised with different plasticisers. This is because glycerol helps maintain the viscosity of the hydrogel and enhances the elongation of the cellulose chain networks, allowing the hydrogel to expand and absorb more water^26^. The hydrogel with these two concentrations, 1.0% (wt/wt) and 1.5% (wt/wt) of glycerol, showed high water uptake capacity, 3562.97% and 4192.17%, respectively. This proves that glycerol at these two concentrations can provide higher flexibility for the polymer chains in the hydrogel’s network. Meanwhile, the cellulose-glycerol-based hydrogel with a concentration of 1.75% (wt/wt) exhibits aless porous surface morphology, as shown in Table 1. This indicates that there is less spacing available for water, as seen in Fig. 2. Consequently, the swelling ratio at 1.75% (wt/wt) returns to a high water uptake capacity due to the increased porosity of the hydrogel. The hydrogels plasticised with NaCMC and PEG both also exhibited high water uptake capacities, reaching 2603.24% and 2672.79%, respectively. However, the cellulose-AA-based hydrogel achieved a water uptake capacity of only 808.3% in this study. From this observation, it is clear that AA, as a plasticiser, does not significantly improve the water uptake capacity of the cellulose hydrogel compared to the other plasticisers. The cellulose-AA-based hydrogel is suspected to have fewer hydrophilic groups than the other hydrogels in this study and weaker flexibility in its cellulose chains. This likely results in a hydrogel with reduced expansion capability when absorbing water. However, cellulose-NaCMC-based hydrogels have a higher swelling ability than cellulose-AA-based hydrogels. As the gelling agent in the formation of hydrogels, NaCMC provides numerous hydrophilic functional groups, such as carboxylic and hydroxyl groups, which facilitate the attachment of water more readily to the hydrogel^13^. Moreover, the water uptake capacity of the hydrogels with a concentration above 1.75% (wt/wt) of NaCMC have weaker water absorption ability, which may be attributed to the decreased viscosity of the hydrogels, limiting the movement of the polymer chains structure^13^. On the contrary, the cellulose-PEG-based hydrogel with a lower amount of PEG exhibits a higher water uptake capacity because fewer unreacted or free PEG chains remain unplasticised with the cellulose chains. When a higher amount of PEG is present, it creates greater steric hindrance, which blocks the cellulose chains and crosslinker from effectively binding to the ends of the PEG molecules, resulting in a reduced water absorption capacity^27^.

**Table 1:**
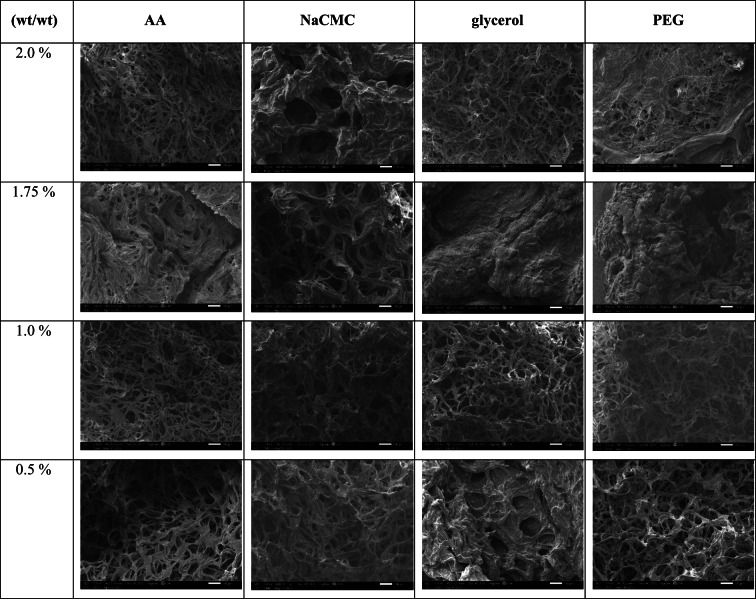
The SEM images of the cellulose hydrogels plasticised with different gelling agents.

### Water retention rate of cellulose-based hydrogels

**Fig. 3:**
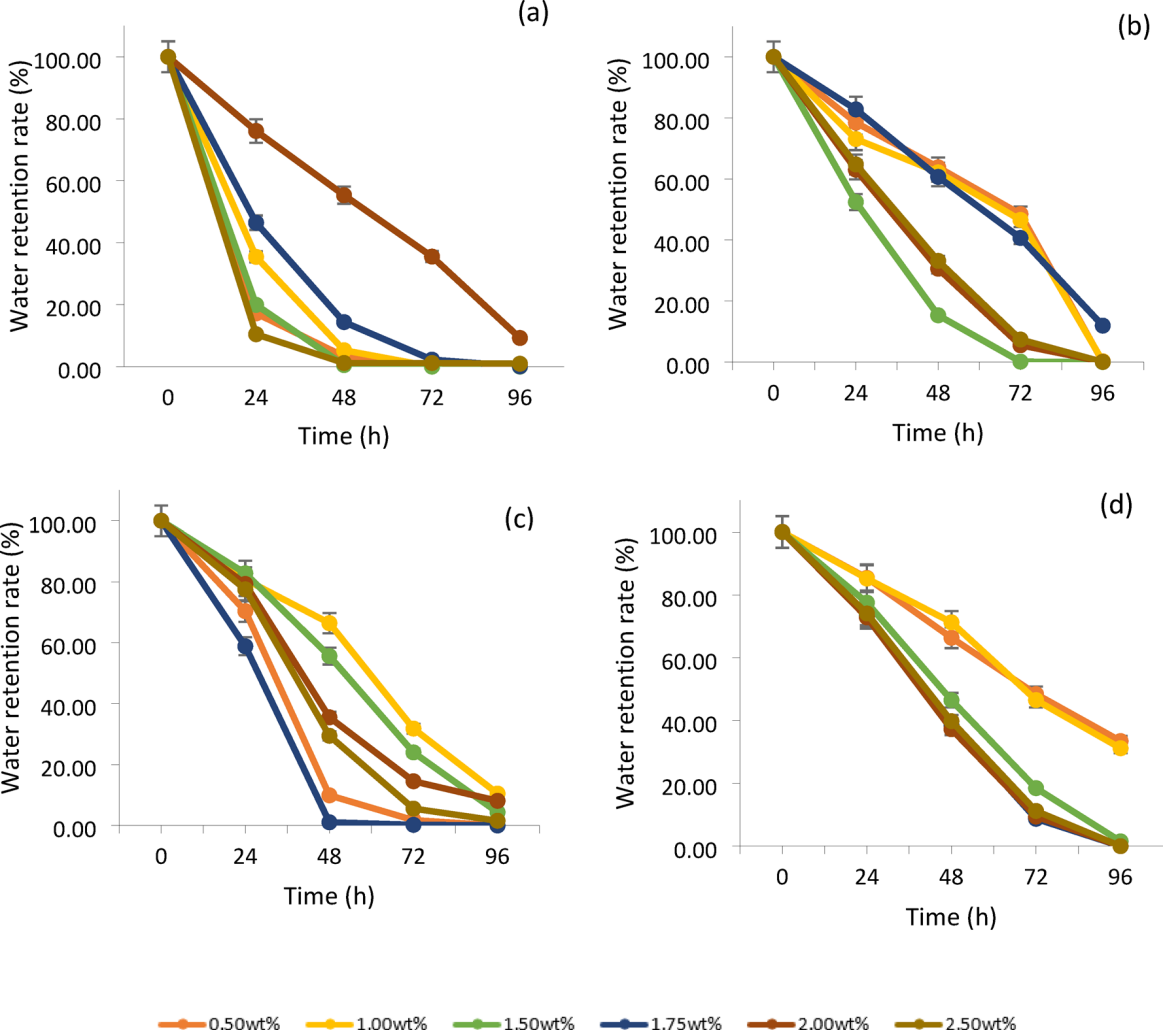
The water retention rate of the cellulose-based hydrogel with different amounts of (**a**) AA; (**b**) NaCMC; (**c**) glycerol; (**d**) PEG.

**Fig. 4:**
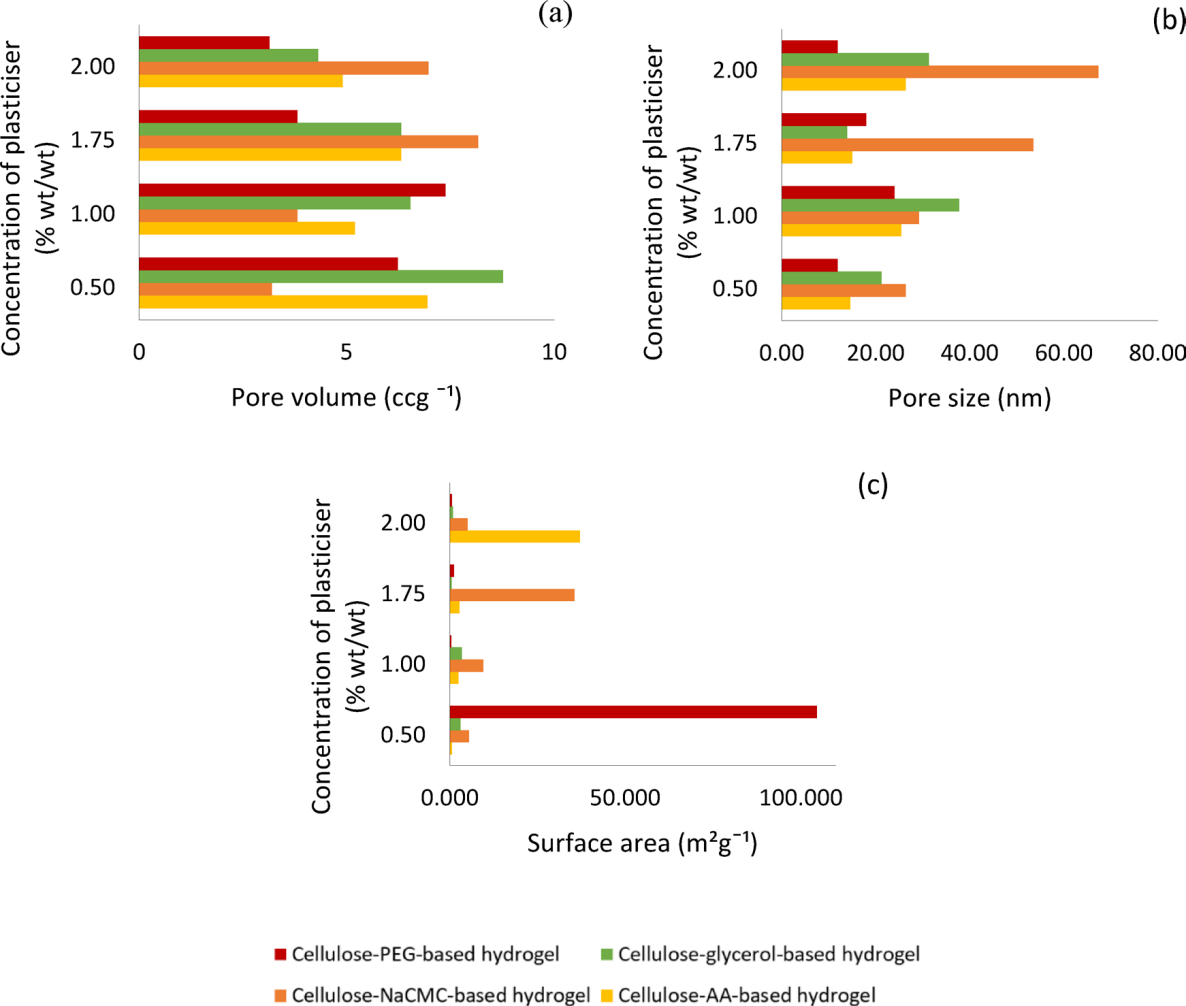
The comparison of the BET data among four different cellulose hydrogels.

The hydrogels shaped with AA, shown in Fig. 3a, demonstrated that the one with 2.0% AA (wt/wt) can hold the most water of all the concentrations tested. The initial water retention rate of the hydrogels with 2.0% (wt/wt) of AA is at 100%, and its water retention rate remained high after being placed in the open air for 24 h, recorded at 76.06%. After 96 h, its water retention rate remained at 9.24%. In addition to this concentration, the water retention rates of hydrogels with other concentrations of AA dropped below 60% after 24 h and were all less than 1% after 96 h. This indicated that the hydrogels with 2.0% (wt/wt) of AA have excellent water retention capability among the other concentrations. When comparing the water retention capacity among all the hydrogels plasticised four different plasticisers at this concentration, the hydrogel with AA and glycerol had 9.24%, and 8.09% of the water remained in the hydrogels after 96 h. However, the hydrogels with NaCMC and PEG at this concentration did not have any water still stored in the structure. This is because the hydrogel containing NaCMC and PEG at this concentration, as shown in Table 1, did not demonstrate better porosity, so it resulted in poor water retention. The hydrogels containing 2.0% (wt/wt) AA and glycerol exhibit numerous small pores and are associated with high swelling ability. This structure helps reduce the amount of water evaporating from the hydrogels. These two characteristics directly enhance the water retention capacity of the hydrogels^3^. However, the hydrogels with glycerol at this concentration had a lower water retention capacity than those with AA. This could be because the pore volume of the glycerol-containing hydrogel at this concentration is smaller than the pore volume of the AA-containing hydrogel, which is only 4.315 ${\text{ccg}}^{ - 1}$ in Fig. 4a. However, the pore volume of the AA-containing hydrogel is 4.900 ${\text{ccg}}^{ - 1}$, indicating that the cellulose-AA-based hydrogel at this concentration has larger spaces for water to fill in. Another reason that the AA-containing hydrogel at this concentration has better retention ability is that it has a smaller average pore size of 26.4 ± 0.3 nm, which can prevent the water trapped in the structure from evaporating from the hydrogel. In contrast, the hydrogel with glycerol has a bigger average pore size of 31.3 ± 0.3 nm in this study.

For the water retention rate of hydrogels with NaCMC, as shown in Fig. 3b, the hydrogels with 1.75% (wt/wt) of NaCMC exhibited better water retention compared to the other cellulose hydrogels with different concentrations of NaCMC. It still had 11.95% of the water in its structure after 96 h, but the rest of the hydrogels could not store the water in their structure over 72 h, although the swelling ratio of hydrogel with 0.5% (wt/wt) and 1.0% (wt/wt) NaCMC are high. The reason that caused the hydrogel with 1.75% (wt/wt) NaCMC to have a higher water retention ability than other concentrations of NaCMC is the elongation of the gel network at this concentration, a large number of carboxylic groups and the presence of Donnan osmotic pressure caused by the presence of the high amount of ${\text{Na}}^{ + }$ ions throughout the hydrogel’s polymeric structure^13^. Besides, the surface morphology of this hydrogel is more porous compared with the other hydrogels at this concentration in Table 1. It has the largest average pore size of 53.50 ± 0.2 nm and denser porous with a pore volume of 8.168 ${\text{ccg}}^{ - 1}$, provided more empty spaces for retaining the water in each pore. This was further confirmed by the fact that the NaCMC with 1.75% (wt/wt) exhibited a high gel fraction and superabsorbent property^13^.

Moreover, the cellulose-glycerol-based hydrogels with 1.00% (wt/wt) glycerol can retain water for a longer time, storing more than 10% of water in its structure even after 96 h in Fig. 3c. The surface morphology of the hydrogel with 1.0wt% glycerol, as shown in Table 1, is more porous with a small size of around 37.74 ± 0.3 nm and a larger pore volume of 6.535 ${\text{ccg}}^{ - 1}$ in its interpenetrating network, resulting in this hydrogel at this concentration being able to store the water in its structure after 96 h. The surface morphologies of the hydrogels with the other three plasticisers at this concentration are shown in Table 1, indicating that the larger pores lead tofaster evaporation of the stored water within the structure. The use of glycerol as a plasticiser at this concentration in hydrogel formation can reduce the density of the hydrogel matrix, allowing for greater movement of the cellulose chains. This results in increased flexibility and malleability of the hydrogel^21^. The water retention rate of the cellulose hydrogel with 1.5% (wt/wt) of glycerol is only 4.32%. It is unable to retain much water after 96 h and starts to dry up after a day, although it has a high swelling ability and larger pore volume (7.380 ${\text{ccg}}^{ - 1}$) in Fig. 4a. This shows that not all hydrogels with high swelling ability can retain water for longer periods. It is suspected that the pores are too large, with an average pore size of 67.13 ± 0.3 nm, which is shown in Fig. 4b, so they can store more water but are not able to prevent the water from evaporating at a low rate. The reason the hydrogel with 1.75% (wt/wt) glycerol has weaker porosity compared to other concentrations may be due to the organization of the polysaccharide polymer molecules. At this concentration, the association of the hydrophobic regions is significantly affected, causing the polymer structure to become more amorphous and reducing the intermolecular strength. The water retention rate of hydrogels with a lower concentration of PEG, specifically 0.5% (wt/wt), showed that 33.47% of the water remained within the hydrogels after 96 h, as depicted in Fig. 3d, resulted in high water retention and high porosity in its surface morphology, as shown in Table 1. Besides high porosity, it also exhibited a greater surface area at around 104.575 m^2^ ${\text{g}}^{ - 1}$ with a smaller pore size (11.92 ± 0.3 nm) and significant pore volume (6.232 ${\text{ccg}}^{ - 1}$) in Fig. 4a, b, c. Thus, this concentration represents the optimal level for producing a highly porous hydrogel. At this concentration, the hydrogel containing AA, NaCMC, and glycerol is unable to retain water due to its lack of numerous pores, as demonstrated in Table 1. Additionally, PEG acts as a plasticiser by reducing intermolecular forces and enhancing the mobility of the polymer chains. The hydroxyl groups in the PEG chains contribute to this effect, resulting in increased moisture content within the hydrogel^28^.

When comparing all the highest water retention rates of each hydrogel, the hydrogel with PEG as a plasticiser demonstrates the highest water retention capacity. This is also evident in its surface area, pore volume, and pore size, where the cellulose-PEG-based hydrogel has the highest surface area at 104.575 m^2^ ${\text{g}}^{ - 1}$ and the smallest average pore size of 11.92 ± 0.3 nm. This small pore size helps prevent water molecules from escaping the hydrogel structure despite its pore volume being only 6.232 ${\text{ccg}}^{ - 1}$. In contrast, the hydrogels with NaCMC and glycerol, while having high water uptake capacity and larger pore volumes, exhibit lower water retention capacity. This is because their average pore sizes are larger—53.50 ± 0.2 nm for the NaCMC hydrogel and 37.74 ± 0.3 nm for the glycerol hydrogel. Thus, the larger the pore size in the hydrogel, the lower its water retention ability. The hydrogel with AA exhibits the weakest water retention capacity compared to the other three hydrogels, as its pore volume is only 4.900 ${\text{ccg}}^{ - 1}$. This smaller pore volume results in weaker water uptake and retention capacity, as there is less space available to store water within the hydrogel.

Our studies demonstrated that cellulose-based hydrogels with various gelling agents, namely 1.75 wt% NaCMC, 2.0 wt% AA, 1.0 wt% glycerol, and 0.5 wt% PEG as its plasticiser, showed excellent water uptake capacity and the higher water retention rate whereby, they kept the water in their structure for the longest time. So, these hydrogels are used in the application part for determining the effect of types of cellulose-based hydrogels on the soil moisture, soil pH, soil electrical conductivity, seed germination of Choy Sum vegetable seeds and the biodegradability of these cellulose-based hydrogels in the loamy soil.

### Assessment as a soil condition

#### Soil moisture of various types of soils

**Table 2:** The water content of the cellulose hydrogel was used in the application of this study.

Types of cellulose-based hydrogel	Swelling ratio (%)	Water retention rate (%)
Cellulose-CMC-based hydrogel	2603.24	11.95
Cellulose-AA-based hydrogel	808.30	9.24
Cellulose-glycerol-based hydrogel	3562.97	10.48
Cellulose-PEG-based hydrogel	2697.49	33.47

This study determined the effect of each cellulose-based hydrogel on soil moisture in four different soil types (sand, topsoil, gley soil, and clayey soil). The soil moisture of all the soil types without any hydrogel, known as the control set, did not increase by remaining at 0% for 15 days. This means external factors like the humidity of the surroundings do not affect the soil moisture level in this study. The water content of all the hydrogels used in the soil moisture test is presented in Table 2.

**Fig. 5:**
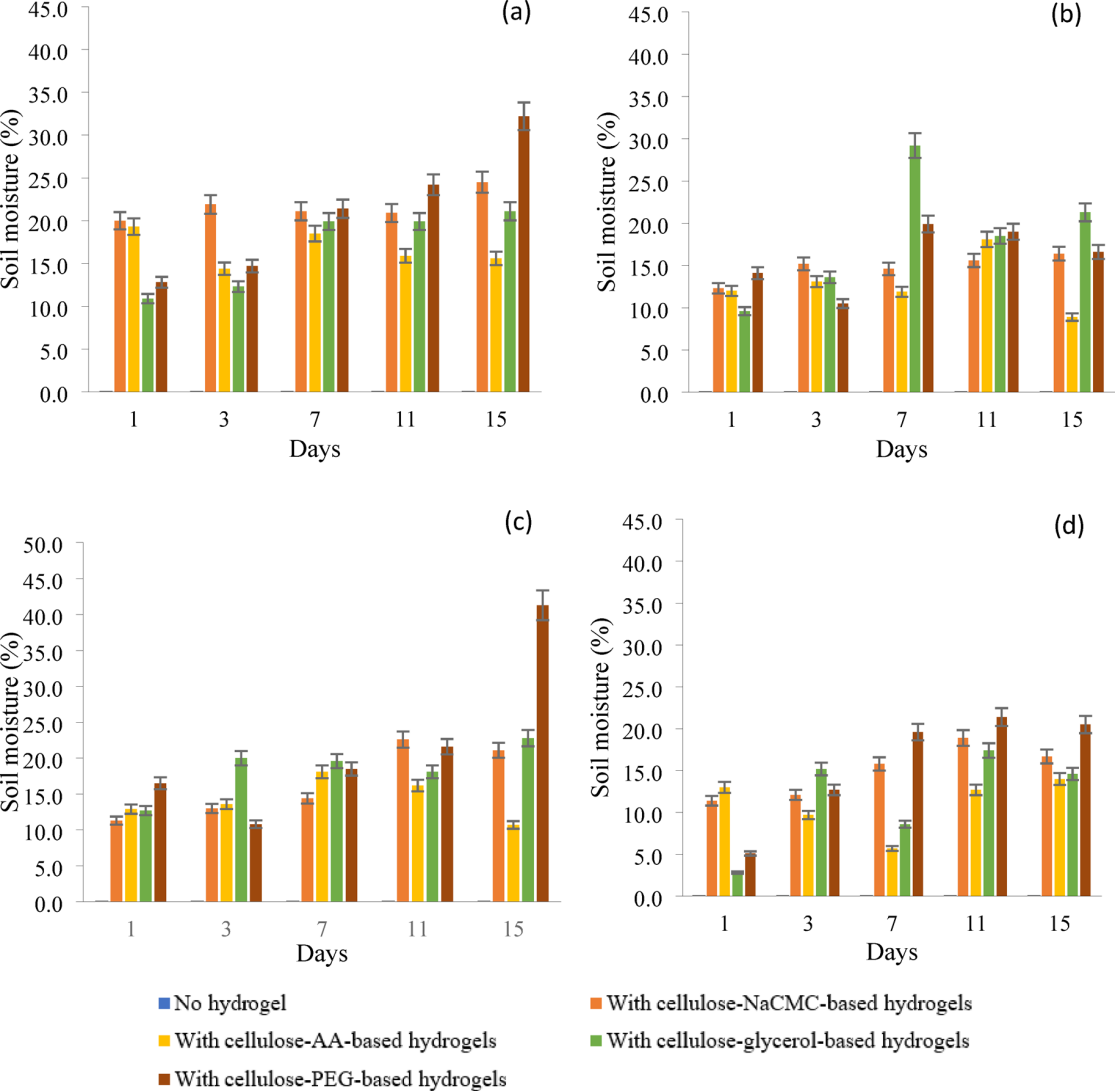
Soil moisture of (**a**) sand, (**b**) topsoil, (**c**) gley soil, and (**d**) clayey soil with cellulose-based hydrogels for 15 days.

Sand is a type of soil rarely used in agriculture, and this has brought enormous challenges due to its low water-holding capacity^29^. However, this study showed a promising enhancement in the moisture content of the sand through various types of cellulose-based hydrogel, as depicted in Fig. 5a. When cellulose hydrogels with PEG and NaCMC were applied to sandy soil, both hydrogels improved the sandy soil’s water-holding capacity. This improvement was due to the hydrogen bonds formed between PEG or NaCMC and the cellulose chains, which introduced more hydrophilic groups into the network structure of the hydrogels^13,30,31^. The cellulose-PEG-based hydrogel exhibited a superior water retention rate and enhanced the soil moisture of the sand compared to other cellulose hydrogels. This was attributed to the significant formation of hydrophilic hydroxyl groups between PEG and cellulose chains, as well as its smaller average pore size. These factors led to an increase in the hydrophilicity of the hydrogel, causing the bound water in the hydrogel to evaporate slowly from the sand. Moreover, the moisture of the sand containing cellulose-glycerol-based hydrogels increased gradually until day 7 and increased slightly by 1.2% on day 15; the soil moisture of the sand with this hydrogel is maintained at around 19.9–21.1% from day 7 until day 15. This shows that the cellulose-glycerol-based hydrogel can provide sufficient water to the sand due to its high water retention ability, keeping the sandy soil hydrated and preventing the water from evaporating from the sand. However, the hydrogel containing AA was less effective in helping the sand retain water than the other hydrogels in this study. This could potentially be attributed to the limited amount of water stored in the cellulose-AA-based hydrogel, which could lead to a slight increase in the sand’s moisture content. Additionally, the hydrogel’s inability to retain water for extended periods may be due to the lack of hydrophilic groups formed between the AA and cellulose chains, thereby failing to maintain moisture levels in the sand. So, the soil moisture of the sand with AA-containing hydrogel dropped to 15.6% until the end of the observation period.

In addition to sand, all the cellulose-based hydrogels improved the soil moisture in the topsoil, although not as effectively as in sand. This is because topsoil is a shallow layer with many empty spaces, making it more prone to water evaporation^32^. The topsoil containing cellulose-glycerol-based hydrogel had the highest soil moisture, reaching 21.3% on day 15 in Fig. 5b. The 7.9% drop in soil moisture from day 7 to day 15 is likely due to the evaporation of water stored in the topsoil. The numerous empty spaces in the topsoil allowed the water provided by the glycerol-based hydrogel to evaporate more quickly, preventing it from being retained for a more extended period. This situation also occurred in topsoil that was treated with cellulose-PEG-based hydrogel. Despite exhibiting a high water retention rate in this study, the hydrogel plasticised with PEG did not significantly increase soil moisture in the topsoil. The soil moisture of the topsoil with this type of hydrogel had risen from 10.5% (day 3) to 19.9% (day 7) but dropped to 16.6% on day 15. This may be due to the water provided by the hydrogel having evaporated, but the hydrogel can still help the topsoil retain 16.6% of the water during day 15. While the cellulose-NaCMC-based hydrogel consistently maintained the soil moisture of the topsoil between 12 and 16% for over 15 days, the properties of the topsoil did not significantly increase or decrease.

The effect of cellulose-based hydrogels on the soil moisture of the gley soil is shown in Fig. 5c. The gley soil with the cellulose-AA-based hydrogel had the lowest soil moisture on day 15 at 10.7%, but it was able to reach 18.1% on day 7. The bound water in this hydrogel had increased the moisture of the gley soil. However, the hydrogel containing AA’s water uptake capacity was insufficient to maintain the moistness of the gley soil for over a week, as the soil’s high concentration of clay particles and organic matter necessitated a higher water intake^33^. Furthermore, the cellulose-PEG-based hydrogel-treated gley soil had the highest soil moisture at 41.3% compared to the other treatments. This is due to its excellent water retention capacity, allowing it to gradually release water over time. Therefore, the clay particles in the soil can hold onto the water that the hydrogel provides, preventing it from evaporating. The cellulose-glycerol-based hydrogel and cellulose-NaCMC-based hydrogel showed similar performance in maintaining soil moisture in the gley soil. By day 15, the gley soil treated with these hydrogels retained 22.8% and 21.1% water, respectively. The study found no significant difference in the water retention ability of both cellulose-based hydrogels, indicating only a small margin in the soil moisture percentage.

Clayey soil is filled with clay particles, which require more water molecules to fill the large surface area of the porosity in the soil system. This necessitates a greater amount of water to fill the void, leading to insufficient water in the hydrogels for the clay particles to absorb^34^ Consequently, the hydrogels plasticised with AA, NaCMC, and glycerol cannot significantly enhance the clayey soil’s moisture content. At 15 days, the soil moisture of those hydrogels was only between 14 and 19% in Fig. 5d. The cellulose-PEG-based hydrogel promoted the highest water availability in the clayed soil, showing 20.5% on day 15 due to its superior water retention compared to other hydrogels.

In conclusion, cellulose-based hydrogels examined in this study can enhance soil moisture in sandy soil, topsoil, gley soil, and clayey soil. However, the cellulose hydrogels plasticised with PEG, NaCMC, and glycerol improve the soil moisture in the four soil types used in this study due to their water uptake capacity and water retention rate.

#### Soil pH of various types of soils

The pH of the soil is also a factor that can influence the growth of the plants. When the soil pH falls within the optimum range of pH 5 to pH 8, most plants, particularly vegetables, can thrive in this range^35,36^. Besides, plants can efficiently uptake the nutrients needed when the soil has the proper pH. So, this study aims to determine whether cellulose-based hydrogels plasticised with NaCMC, AA, glycerol, and PEG can increase the soil pH and maintain it for 15 days.

After mixing 50% cellulose-based hydrogels with each soil type in the planting bags, each soil type with the hydrogels consistently increased and maintained a pH of 5 and above after day 3. It is believed that the increase in soil moisture directly influences the soil’s pH value, as evidenced by the improvement in soil moisture after day 1, leading to a consistent rise in soil pH. After day 3, the pH of the cellulose-glycerol-based hydrogel on the gley soil continued to rise, reaching pH 7.77 on day 15. The formation of a diffusion-driven mechanism between the exterior and interior of the cellulose-glycerol-based hydrogel may have contributed to the slow release of water. Hence, the soil moisture increased slowly, and the pH value also increased slowly^37,38^. Besides, the gley soil contains a high amount of clay particles and organic matter, causing it to trap and hold more water inside, increasing the pH value over 15 days. The presence of hydrogel in the soil can increase the soil pH effectively^39^. Moreover, the pH of the gley and clayed soil initially stood at 4.58 and 5.12, respectively, but it gradually increased to slightly alkaline in all conditions when treated with hydrogel. On the contrary, after 3 days, the pH value of all the sand that contained hydrogels rose from pH 7.4 to pH 8 and above. This suggests that the presence of cellulose hydrogels can elevate the pH value of the sand in response to an increase in soil moisture, thereby establishing a direct correlation between the sand’s moisture content and its pH. Before adding the hydrogels, the pH value of the topsoil was pH 5. After adding the hydrogels, all the pH values increased to pH 6.53 (with cellulose-NaCMC-based hydrogels), pH 5.63 (with cellulose-AA-based hydrogels), pH 7.77 (with cellulose-glycerol-based hydrogels), and pH 7.84 (with cellulose-PEG-based hydrogels) by day 15. All the topsoil pH values increased under all conditions, transitioning from an acidic pH to a slightly alkaline or alkaline pH, with the exception of the condition where no hydrogel was added.

#### Soil electrical conductivity of various types of soils

The soil electrical conductivity is also known as salinity, is an index to measure the number of salts present in the soil and it is related to soil properties that directly affect crop productivity^40^. The effect of the various cellulose-based hydrogels on the salinity of each soil type is important because this can determine whether those selected hydrogels can improve the salinity of the sand, topsoil, gley soil and clayey soil. The soil electrical conductivities of the sand, topsoil, gley soil, and clayey soil of the control set in this study are 63.9 µs/cm, 44.2 µs/cm, 55.0 µs/cm, and 113.9 µs/cm. All the salinity on the control sets was kept constant over 15 days and guaranteed that no external factors like temperature or humidity affected the results of each soil type during the experimental period.

Regarding the effect of the hydrogels on the salinity of the sand, the cellulose-NaCMC-based hydrogels caused a continuous increase in electrical conductivity over the 15-day period. By day 15, the sand treated with this hydrogel exhibited the highest salinity among all the cellulose-based hydrogels, reaching 803.3 µS/cm. In contrast, the other hydrogels plasticised with AA, glycerol, and PEG showed similar salinity levels, remaining below 260 µS/cm. This observation indicates that the cellulose-NaCMC-based hydrogel increased 739.4 µs/cm of the salinity of the sand. However, the other hydrogels showed only a gradual increase in salinity. This may be due to hydrogel with NaCMC having the presence of the Na + ions that roamed freely in the hydrogel’s structure; the presence of the cation will increase the electrical conductivity of the sand^13,41^. In addition to sand, the cellulose-PEG-based hydrogels exhibited the highest electrical conductivity in topsoil (689.7 µs/cm), gley soil (446.7 µs/cm), and clayed soil (386.7 µs/cm). following this, the cellulose-glycerol-based hydrogels showed higher salinity in topsoil (572.0 µs/cm), gley soil (338.0 µs/cm), and clayed soil (336.3 µs/cm). The likely reason that the hydrogel with PEG and glycerol can improve the electrical conductivity of gley soil and clayey soil is that these hydrogels help minimise the leaching loss of essential cations from the soil.

In conclusion, the salinity results of each soil type indicated that the cellulose-based hydrogel plasticised with NaCMC, glycerol, and PEG can improve the electrical conductivity of sandy soil, topsoil, gley soil, and clayed soil.

### Seed germination assay

Forming a soilless condition with the hydrogel is an effective option for seed germination. This was demonstrated in our previous study which showed that the cellulose-based hydrogel plasticised with NaCMC had a better performance on germinating the paddy seeds with soilless condition compared with germinating on the soils^42^. To further study the effectiveness of cellulose-based hydrogels on germinating the common vegetable like Choy Sum vegetable, this study will demonstrate and compare the efficacy of types of cellulose-based hydrogel plasticised with alginic acid (AA), sodium carboxymethyl cellulose (NaCMC), glycerol and polyethylene glycol (PEG) on the seed germination of Choy Sum vegetable seeds. The results of the seed germination on different types of cellulose-based hydrogels were compared with one another, as well as with the result of the seed germination without any hydrogel.

The seed germination rate of the Choy Sum vegetable seeds on the cotton (without hydrogel) watered daily is only 27%, with a germination value of just 2.25%, as shown in Table 3. The seeds germinated without hydrogel and without daily watering did not result in any seeds germinating on the cotton. Therefore, the seeds germinated on the cotton need to be watered daily. Without regular watering, the water will evaporate, leaving insufficient moisture to support seed germination. The traditional method of germinating seeds on cotton is insufficient to support germination without water or with inadequate water supply. However, most seeds germinated successfully on the swollen cellulose-based hydrogels, even when no water was provided during the germination period.

**Table 3:** The germination parameters of the Choy Sum vegetable seeds during the observation period under different treatments.

Treatment	Germination (%)	Mean daily germination (%)	Peak value of germination (%)	Energy period (Days)	Germination value (%)
Germination parameters					
No hydrogel	27.00	3.57	4.00	7	2.25
Cellulose-AA-based hydrogel	40.00	4.29	5.60	7	4.58
Cellulose-NaCMC-based hydrogel	51.00	5.14	5.67	7	5.28
Cellulose-glycerol-based hydrogel	65.00	8.71	11.40	7	16.91
Cellulose-PEG-based hydrogel	49.00	6.29	6.29	7	6.89

Based on the results in Table 3, 65% of the seeds germinated on the cellulose-glycerol-based hydrogel, followed by 51% on the cellulose-NaCMC-based hydrogel, and 49% on the cellulose-PEG-based hydrogel. The cellulose-AA-based hydrogel had the lowest seed germination rate among all the cellulose hydrogels in this study at 40%. There was no water supply to the seeds that germinated on all the cellulose-based hydrogels. Compared to the seed germination of Choy Sum vegetables in the no-hydrogel and daily watering condition, showed that most of the Choy Sum vegetable seeds on these hydrogels successfully germinated, with the seed germination rate at 40% or higher. All treatments in this study were statistically significant, with a p-value of less than 0.05. This may be due to the water-retaining capacity of these hydrogels in their structures, which provide the sufficient moisture needed for seed germination. With cellulose hydrogels, there is no need to water the seeds during the germination period, as the hydrogels retain water and provide soilless conditions that still support seed germination. Moreover, the cellulose-glycerol-based hydrogel had the highest seed germination rate at 61% compared to the seed germination rate among all the selected cellulose hydrogels. This cellulose hydrogel can germinate more seeds because of its water retention ability, as it contains a large number of hydrophilic groups in its structure and continuously provides water to all the seeds^43^.

According to the seed germination value and the mean daily germination percentage of all the conditions in Table 3, the hydrogel with glycerol as its plasticiser had the greatest seed germination value of 16.91% and exhibited the earliest germination, with the most seeds starting to germinate on day 3. The peak value of the daily germination rate is the maximum quotient of the daily germination rate of the Choy Sum seeds on the cellulose-glycerol-based hydrogel during the observation period. This demonstrates that the seeds that germinated on cellulose-glycerol-based hydrogel are earlier and faster as the moisture provided by the hydrogel improves the metabolic activity of the seeds and causes the improvement in the germination process with earlier and better germination^44,45^. On the other hand, although the germination value of the rest of the cellulose hydrogels plasticised with NaCMC, PEG, and AA are between 4.5 and 6.9%, they also promoted seed germination, whereby the seeds germinated on day 3. These hydrogels can improve the seed germination process compared to the seeds that germinate on cotton without any hydrogel because the water uptake capacities of those hydrogels are the main factor that promotes the earlier seed germination process.

### Biodegradability of cellulose-based hydrogels

This study evaluated the biodegradability of each cellulose-based hydrogel plasticised with four plasticisers in the common garden soil known as loamy soil. In this study, the cellulose hydrogels containing NaCMC and AA had already lost their weight by over 60% after 14 days, around 62%, but the weight loss of the cellulose-glycerol-based hydrogels and cellulose-PEG-based hydrogels are not more than 20% which are 18.99% and 6.14%, respectively. After 28 days, the weight loss of the hydrogels with glycerol and PEG had reached 75.49% and 71.77%, which had composted over 60%. This showed that the cellulose hydrogels with NaCMC and AA have faster biodegradability than the other two cellulose hydrogels. However, the starting materials and the method used for the formation of the hydrogel are the same. This may be due to NaCMC and AA not causing the crosslinking density of the cellulose hydrogels to become strong enough, and these two plasticisers belong to the biopolymer, producing the polymer that is easy to compost when buried in the loamy soil^30,46^. Moreover, the hydrogels containing NaCMC and AA had composted up to 96% of the weight loss after being burned in the loamy soil for 60 days. Meanwhile, the cellulose hydrogels contained glycerol and PEG and only composted up to 87% of the weight lost after 60 days. The reason that the cellulose-PEG-based hydrogels and the cellulose-glycerol-based hydrogels are composted at a slower rate is both of these plasticisers increase the crosslink density of the hydrogel and enhance the elasticity of the hydrogel, and its elasticity slows down the degradation process^47,48^. However, all types of cellulose-based hydrogels had a high degree of biodegradability and all the degradation of the hydrogels reached 85%. This finding is also proven by Tefera et al.^39^. This may be due to the glycosidic bonds present in the polysaccharide chains in the cellulose fibres that promote enzymatic degradation by the common enzymes that are found in garden soil like amylase^49^.

## Conclusion

The use of different plasticisers in the formation of cellulose-based hydrogels influenced various characteristics of the hydrogels, including their surface morphology, chemical bonding, water absorption capacity, and water retention ability. The cellulose-based hydrogels containing NaCMC, glycerol, and PEG have high water uptake and retention capacity, enhancing the soil moisture, pH and electrical conductivity in various soil types. Moreover, the seed germination rate of the Choy Sum vegetable on the cellulose-glycerol-based hydrogels was excellent, reaching 65%, compared to just 27% for the seeds germinated without any hydrogel. All cellulose hydrogels were decomposed and lost up to 87% of their weight after 60 days in the loamy soil.

## Data Availability

The data supporting this study’s findings are available from the corresponding author upon reasonable request.
